# Magnetic Resonance Imaging Findings of Intraspinal Tuberculoma in Children

**DOI:** 10.3389/fneur.2022.936837

**Published:** 2022-08-02

**Authors:** Yirui Zhou, Yong Qin, Tong Mu, Helin Zheng, Jinhua Cai

**Affiliations:** ^1^Department of Radiology, National Clinical Research Center for Child Health and Disorders, Ministry of Education Key Laboratory of Child Development and Disorders, Chongqing Key Laboratory of Pediatrics, Children's Hospital of Chongqing Medical University, Chongqing, China; ^2^Department of Endocrinology and Metabolism, West China Hospital, Sichuan University, Chengdu, China

**Keywords:** tuberculoma, intraspinal, magnetic resonance imaging, children, paradoxical response

## Abstract

**Background and Purpose:**

Intraspinal tuberculoma is a rare disease in children, and its imaging findings have been described in only a few case reports. This study aimed to investigate the magnetic resonance imaging (MRI) features of pediatric intraspinal tuberculoma and to explore the possible pathogenesis of the disease.

**Materials and Methods:**

The clinical and MRI data of 24 child patients with intraspinal tuberculoma (such as 6 cases of intramedullary tuberculoma, 8 cases of intradural extramedullary tuberculoma, and 10 cases of epidural tuberculoma) were retrospectively analyzed. All patients underwent plain and contrast-enhanced MR scans. The diagnosis was confirmed by surgical pathology or by antituberculous treatment and follow-up data.

**Results:**

Intramedullary tuberculoma had a round shape, while intradural extramedullary tuberculoma and epidural tuberculoma presented long-fusiform or en plaque shapes. Regarding MRI signals, intramedullary tuberculoma and extramedullary tuberculoma were mainly isointense on T1-weighted imaging (T1WI) and hypointense or isointense on T2WI. Rim enhancement was observed in intramedullary tuberculoma, and marked homogeneous enhancement was dominant in extramedullary tuberculoma. Ten (10/24) tuberculomas occurred during antituberculous therapy, with intradural extramedullary tuberculoma accounting for 7 cases (7/8), which was significantly more frequent than intramedullary tuberculoma (1/6) or epidural tuberculoma (2/10).

**Conclusion:**

MRI is important in the diagnosis of intraspinal tuberculoma, which is characterized by isointensity on T1WI, isointensity, or hypointensity on T2WI, and rim or obvious homogeneous enhancement. Some intraspinal tuberculomas, especially intradural extramedullary tuberculomas, might be associated with the “paradoxical response” mechanism during the tuberculosis treatment.

## Introduction

Tuberculosis, a chronic infectious disease caused by *Mycobacterium tuberculosis* (MTB), is one of the major causes of death worldwide, still has a relatively high incidence rate in developing countries, and imposes tremendous challenges to social economy and public health (1, 2). Pulmonary tuberculosis is the most common type of systemic tuberculosis infection. Central nervous system (CNS) tuberculosis mainly results from a primary MTB-infected pulmonary lesion that disseminates to the brain, spinal cord parenchyma, meninges, and surrounding tissues *via* the blood. CNS tuberculosis is relatively rare, accounting for 1–5% of all tuberculosis types, but it has a poor prognosis and fairly high fatality and disability rates (3–6). Intraspinal tuberculosis is even rarer in clinical practice, mostly manifesting as tuberculous spinal meningitis and tuberculoma. Intraspinal tuberculoma can be further divided into intramedullary tuberculoma, intradural extramedullary tuberculoma and epidural tuberculoma based on the location, which is difficult to identify and diagnose in the early stage because of the insidious onset, non-specific clinical manifestations, and unfavorable sensitivity of laboratory examinations (7–11).

Magnetic resonance imaging (MRI) is an irreplaceable modality in the diagnosis of intraspinal lesions due to such advantages as high resolution of soft tissues and no radiation (12–15). Some MRI findings of intraspinal tuberculoma have been described in the literature, most of which corresponded to individual cases (16–21). Therefore, the MRI findings of 24 children with intraspinal tuberculoma verified by surgery or clinical follow-up were analyzed in this paper, and the potential pathogenesis of intraspinal tuberculoma at different locations was explored using clinical data, thereby offering an imaging basis for the early diagnosis and treatment of the disease.

## Materials and Methods

### Ethics Statement

The study was conducted in compliance with the ethics principles of the Declaration of Helsinki and approved by the Institutional Ethics Committee of Children's Hospital Affiliated to Chongqing Medical University. Due to the retrospective nature of the study, the need for informed consent was waived.

### Case Data

A total of 24 children who were diagnosed with intraspinal tuberculoma from January 2008 to February 2022 and had complete clinical and imaging data were enrolled, such as 6 cases of intramedullary tuberculoma, 8 cases of intradural extramedullary tuberculoma, and 10 cases of epidural tuberculoma. There were 18 cases confirmed by surgical pathology and 6 cases confirmed by clinical and laboratory examinations, antituberculous therapy and follow-up. Among the 24 children, 15 were men and 9 were women; their ages ranged from 3 to 18 years, with an average of 11.82 years (Table 1).

**Table 1:** Clinical and MRI data of 24 patients with intraspinal tuberculoma.

**Case No**.	**Age (years)**	**Sex**	**Symptoms/signs**	**Location**	**T1WI (intense)**	**T2WI (intense)**	**Contrast enhancement**	**Therapy modes**	**Outcome**
1	11.6	M	LL paralysis	Intra	Iso-	Hypo-	Rim	Surgery	Cured
2	9.9	M	UL and LL paralysis/sensory dysfunction	Intra	Iso-	Hypo-	Rim	Anti-TB medicine	Cured
3	15.0	F	LL sensory dysfunction	Intra	Hyper-	Hypo-	Rim	Surgery	Cured
4	12.9	M	LL paralysis/sensory dysfunction	Intra	Iso-	Hypo-	Rim	Surgery	Cured
5	3.0	F	LL paralysis/sensory dysfunction	Intra	Hyper-	Hypo-	Rim	Anti-TB medicine	Cured
6	13.2	M	LL paralysis/sensory dysfunction	Intra	Iso-	Hypo-	Rim	Anti-TB medicine	Cured
6	14.7	M	LL paralysis	IE	Hypo-	Hyper-	Homogeneous	Surgery	Cured
7	16.5	F	UL and LL paralysis/sensory dysfunction/urinary dysfunction	IE	Hypo-	Hypo-	Homogeneous	Surgery	Improved
8	12.3	M	LL paralysis/sensory dysfunction/urinary dysfunction	IE	Iso-	Hyper-	Homogeneous	Surgery	Improved
8	10.4	M	LL paralysis/sensory dysfunction	IE	Iso-	Hypo-	Homogeneous	Surgery	Cured
10	14.5	F	UL and LL paralysis/sensory dysfunction/urinary dysfunction	IE	Iso-	Iso-	Heterogeneous	Surgery	Improved
11	10.1	M	LL paralysis/sensory dysfunction	IE	Iso-	Iso-	Homogeneous	Surgery	Cured
12	8.8	F	LL paralysis/sensory dysfunction/urinary dysfunction	IE	Iso-	Iso-	Heterogeneous	Surgery	Improved
13	15.7	M	LL paralysis/sensory dysfunction	IE	Iso-	Iso-	Homogeneous	Surgery	Cured
14	10.4	M	LL paralysis/sensory dysfunction	Epi	Hypo-	Iso-	Rim	Surgery	Cured
15	15.5	M	LL paralysis	Epi	Hypo-	Hyper-	Homogeneous	Surgery	Cured
16	18.0	F	LL paralysis/sensory dysfunction	Epi	Hypo-	Hyper-	Homogeneous	Surgery	Cured
17	10.2	F	UL and LL paralysis/urinary dysfunction	Epi	Iso-	Hypo-	Homogeneous	Surgery	Improved
18	5.4	M	LL sensory dysfunction	Epi	Iso-	Iso-	Homogeneous	Anti-TB medicine	Cured
19	13.8	M	LL paralysis/sensory dysfunction/urinary dysfunction	Epi	Iso-	Iso-	Homogeneous	Surgery	Improved
20	12.0	F	LL paralysis/sensory dysfunction	Epi	Iso-	Hypo-	Rim	Surgery	Cured
21	12.9	M	UL and LL paralysis/sensory dysfunction	Epi	Iso-	Hypo-	Homogeneous	Anti-TB medicine	Improved
22	8.2	M	LL paralysis/sensory dysfunction/urinary dysfunction	Epi	Iso-	Hypo-	Homogeneous	Surgery	Cured
24	8.6	F	LL sensory dysfunction	Epi	Iso-	Hypo-	Homogeneous	Anti-TB medicine	Cured

*M, male; F, female; UL, upper limbs; LL, lower limbs; Intra, intramedullary; IE, intradural extramedullary; Epi, epidural; Hypo-, hypointense; Iso-, isointense; Hyper-, hyperintense; TB, tuberculosis*.

Among all 24 cases, 14 children were complicated with pulmonary tuberculosis, 8 children were complicated with tuberculous meningitis, 8 children had tuberculosis of adjacent vertebra or the appendix, and 5 children had comorbid tuberculosis of the liver, spleen, intestine, or other organs. The clinical manifestations were dominated by various degrees of compression symptoms of the spinal cord or spinal nerve root. The initial manifestations were mild paralysis and/or sensory function decline of the lower limbs; 5 cases were accompanied by mild paralysis and/or sensory function decline of the left upper limb, and 7 cases were complicated with constantly aggravated urinary incontinence or retention (Table 1).

In 10 children, compression symptoms of the spinal cord or spinal nerve root emerged within 1–9 months after treatment with antituberculosis drugs, such as 4 cases at 1–3 months, 5 cases at 4–6 months, and 1 case at 7–9 months. The duration from symptoms onset to lesion detection by MRI was 1–14 days, with a median of 8 days.

The lesions detected by MRI were resected *via* surgery in 18 cases, and post-operative treatment with antituberculosis drugs was conducted for 3–9 months. The remaining 6 cases were merely treated with routine antituberculosis drugs. The clinical manifestations of all patients were recovered to different extents following operation and/or antituberculous therapy. During 3–9 months of follow-up, there were 7 cases of mild paralysis of the lower limbs, which was ameliorated to various degrees compared with that before operation, and the 17 remaining children exhibited completely restored motor and sensory functions of the lower limbs.

### MRI Examination

All patients were imaged by a 1.5 T (Signa HDxt, GE, USA) or a 3.0 T (Achieva, Philips, Holland) MR scanner configured with standard spinal coils. Specifically, contrast-enhanced scans for spin-echo T1-weighted imaging (T1WI) and fast spin-echo T2WI and T1WI were performed for the spine in the sagittal and axial planes. The scan parameters were set as follows: T1WI: repetition time (TR) = 400–450 ms and time of echo (TE) = 8–12 ms; T2WI: TR = 3,000–5,000 ms, TE = 90–120 ms; slice thickness = 3–5 mm; gap = 2–3 mm; matrix = 256 × 256; and excitation frequency = 2–4 times. Magnevist (Gd-DT-PA) at a conventional dose (0.1 mmol/kg) was intravenously injected as a bolus during contrast-enhanced scanning. Uncooperative patients younger than 5 years of age were orally administered 10% chloral hydrate at 3–5 ml/kg for sedation.

### Analysis of MRI Images

The MRI images of all patients were independently reviewed by two neuroradiologists with 10–30 years of experience. The image features to be evaluated included: lesion location (intramedullary, intradural, or epidural; cervical, thoracic, or lumbosacral segments of the spine), lesion shape (round, round-like, fusiform, strip-like, en plaque or irregular), signal intensity on T1WI and T2WI (hypointense, isointense, or hyperintense relative to the spinal cord; homogeneous or heterogeneous), and enhancement pattern (mild, moderate, or marked; homogeneous, heterogeneous, or rim-like). In cases of disagreement, a consensus was reached through discussion and consultation.

### Statistical Analysis

The incidence of intraspinal tuberculoma during antituberculous therapy among the three groups was statistically analyzed using Fisher's exact test. The *p-*values <0.05 (two-sided) were considered indicative of a statistically significant difference. The SPSS version 17.0 (IBM Corporation, Armonk, NY, United States) was applied for statistical analyses.

## Results

### Magnetic Resonance Imaging Findings

**Figure 1:**
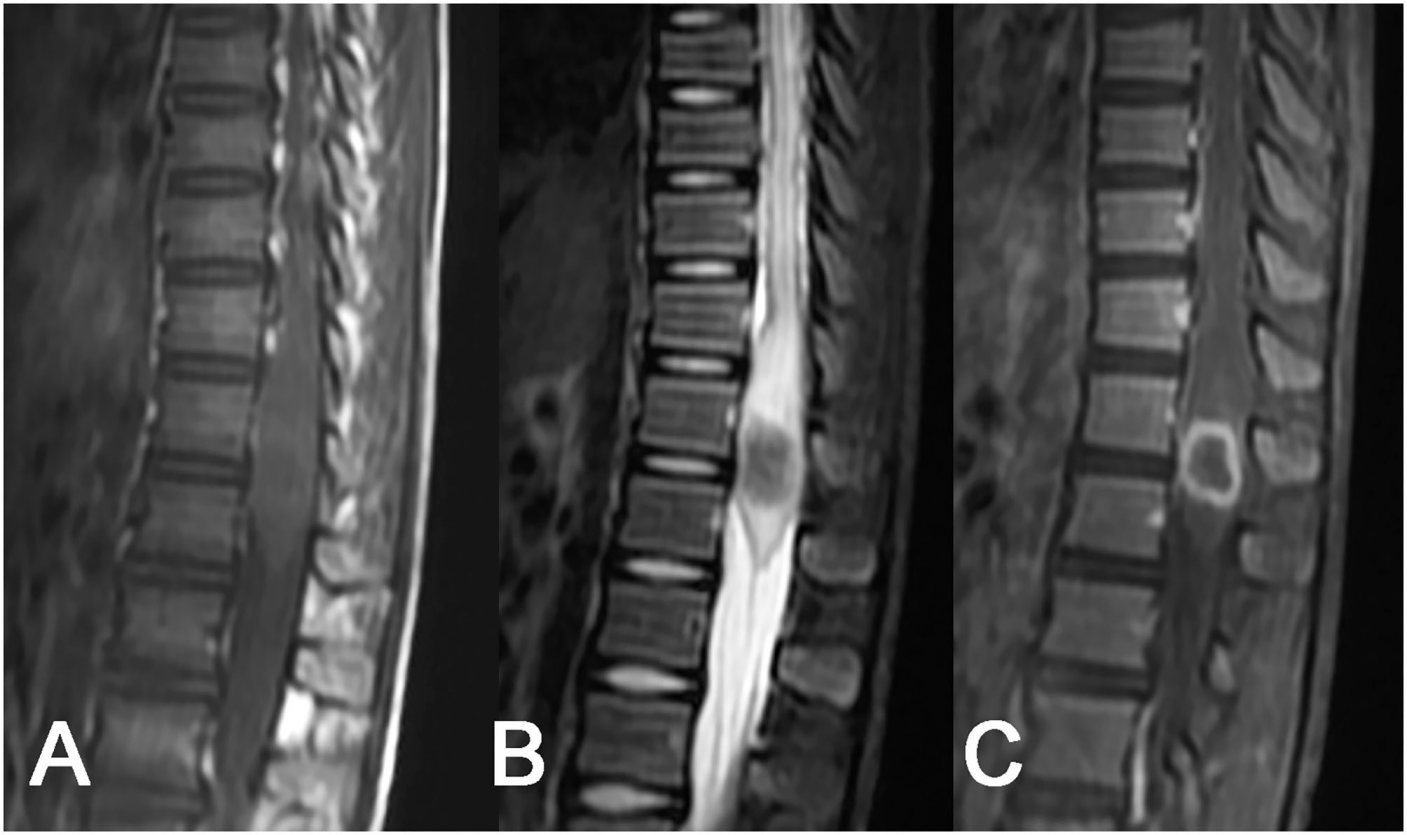
Tuberculoma of the conus medullaris. A slightly enlarged round-like lesion at the conus medullaris: **(A)** isointense on T1WI, **(B)** hypointense on T2WI, with poorly defined margins and swelling of the adjacent spinal cord, and **(C)** homogeneous rim enhancement after contrast enhancement.

Among 6 cases of intramedullary tuberculoma, 4 cases were located in the cervical spinal cord, 1 case was in the thoracic spinal cord, and 1 case was detected in the lumbar spinal cord. Intramedullary tuberculoma presented a round or round-like shape, with no or mild enlargement at corresponding segments of the spinal cord and with different degrees of edema of the adjacent spinal cord. Intramedullary tuberculoma was isointense or slight hyperintense on T1WI, with poorly defined margins (Figure 1A). Hypointense on T2WI was observed, with or without slight hyperintense on the margins (Figure 1B). Apparent rim enhancement was observed on the contrast-enhanced images (Figure 1C; Table 1).

**Figure 2:**
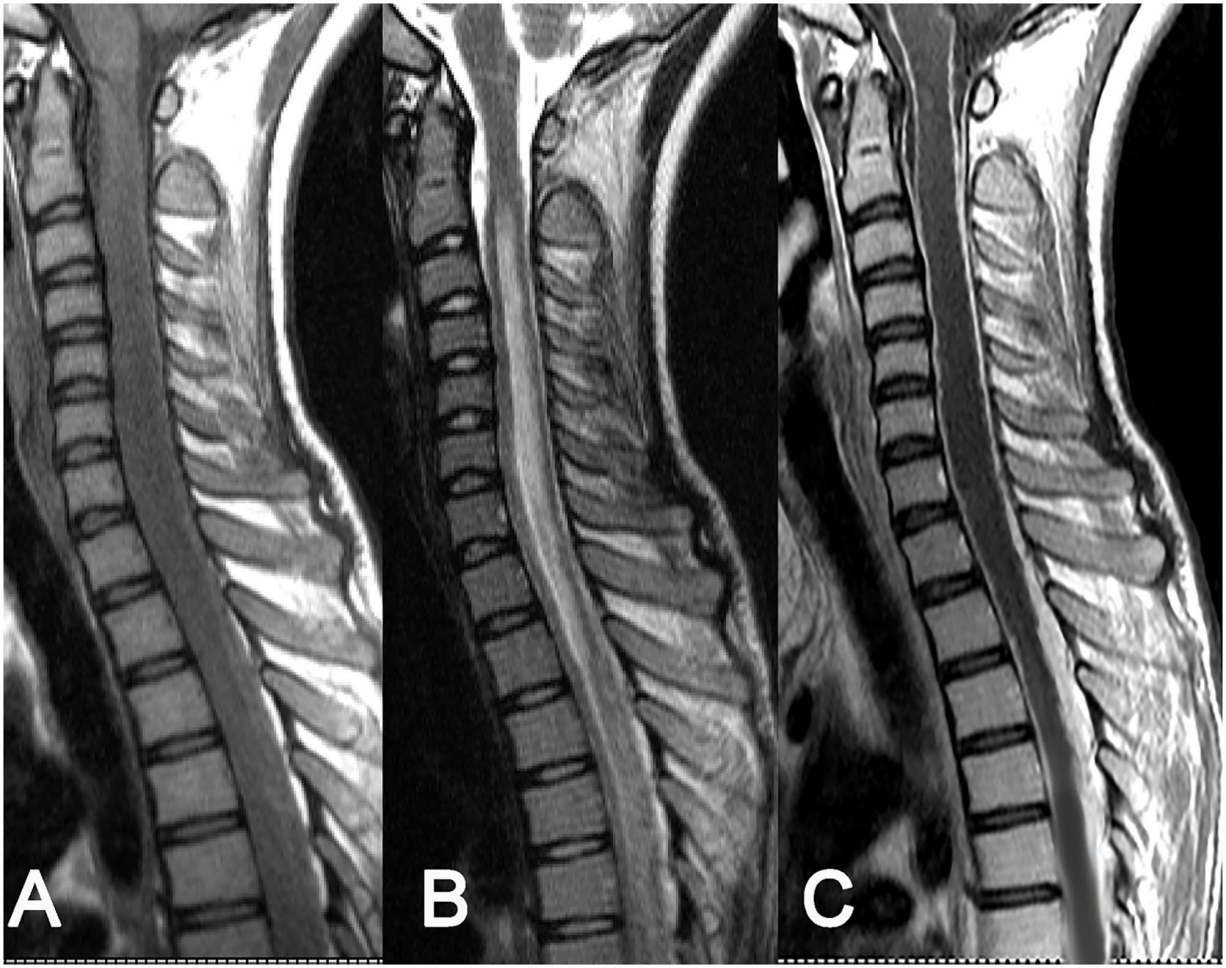
Intradural extramedullary tuberculoma of the thoracic spinal cord. A lesion having a longitudinal fusiform shape: **(A)** isointense on T1WI, **(B)** isointense on T2WI, and **(C)** obvious homogeneous enhancement after contrast enhancement, accompanied by diffuse enhancement of spinal meninges.

**Figure 3:**
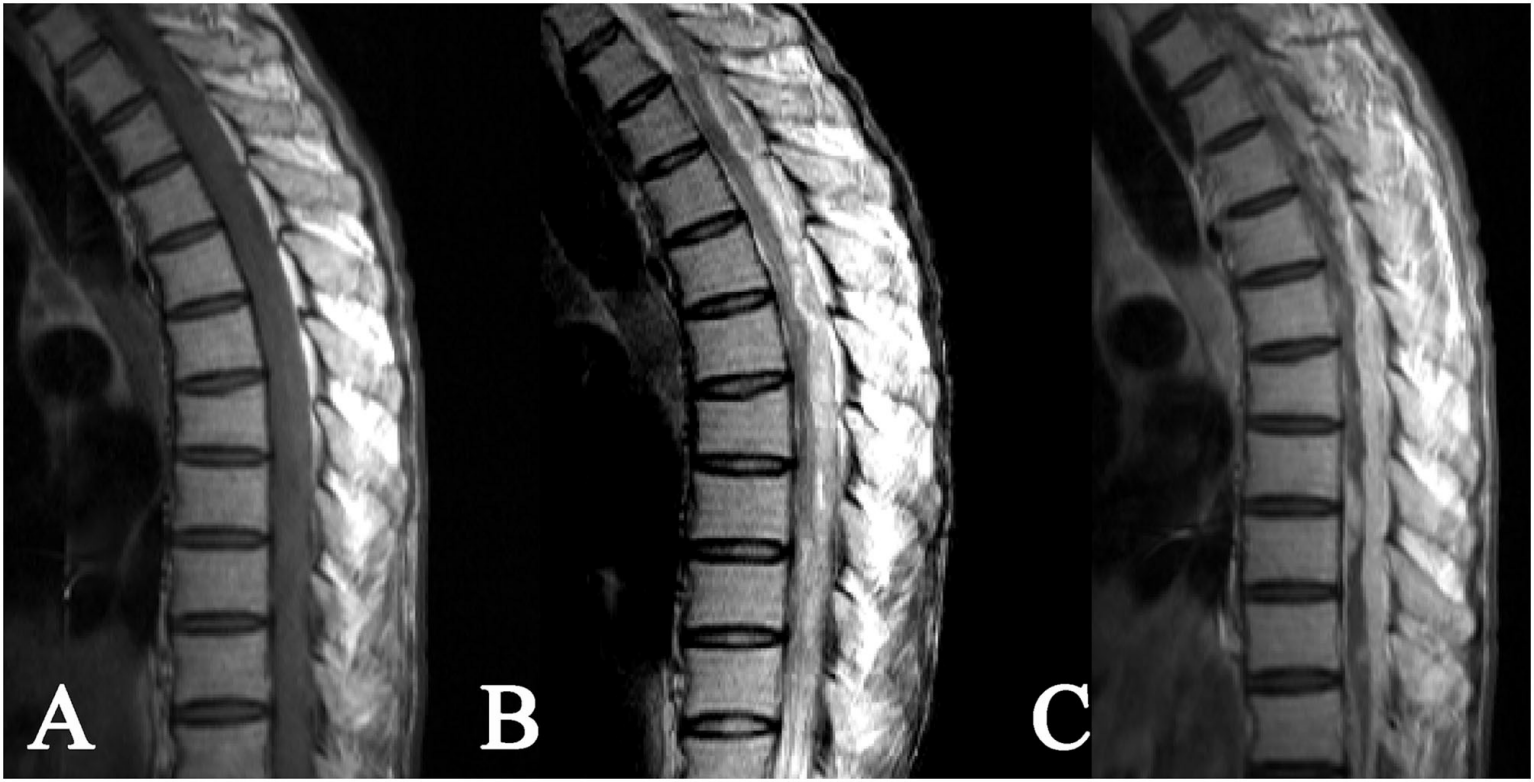
Intradural extramedullary tuberculoma of thoracolumbar spinal cord. A lesion having a longitudinal long-fusiform shape: **(A)** isointense on T1WI, **(B)** hyperintense on T2WI, and **(C)** apparent heterogeneous enhancement after contrast enhancement, showing a narrowed adjacent spinal cord due to compression.

As for the 8 cases of intradural extramedullary tuberculoma, there was 1 case in the cervical spinal cord, 4 cases in the thoracic spinal cord, 1 case in the lumbar spinal cord, 1 case at the cervicothoracic junction, and 1 case at the thoracolumbar junction. The lesions showed a longitudinal course along the posterolateral or posterior side of the spinal cord and had long-fusiform shapes. The longitudinal length of the lesion covered 2–4 vertebrae in 5 cases, 4–8 vertebrae in 2 cases, and more than 8 vertebrae in only 1 case. The corresponding segments of the spinal cord were compressed to different extents, and 1 case of local spinal cord edema was observed. The MRI signal features of tuberculoma involved slight hypointense or isointense on T1WI (Figures 2A, 3A), hypointense, isointense, or hyperintense on T2WI (Figures 2B, 3B). On contrast-enhanced images, marked enhancement was observed in all 8 lesions, 7 of which showed homogeneous enhancement (Figures 2C, 3C), while 1 lesion showed heterogeneous enhancement with patchy unenhanced regions (Figures 2C, 3C; Table 1).

**Figure 4:**
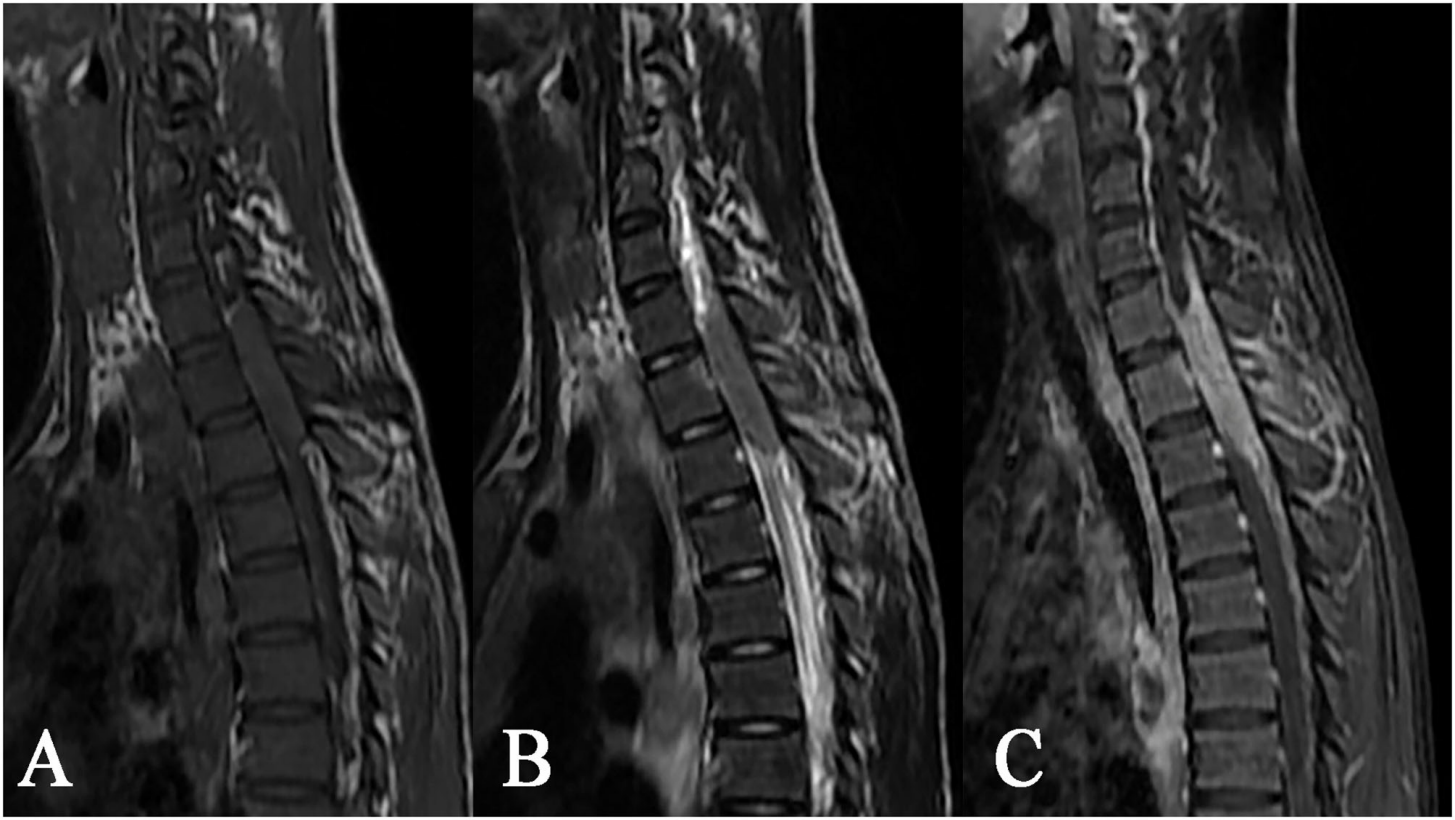
Epidural tuberculoma at the cervicothoracic junction. A lesion having a longitudinal fusiform shape: **(A)** isointense on T1WI, **(B)** isointense on T2WI, and **(C)** distinct homogeneous enhancement after contrast enhancement, showing extension to adjacent spinal meninges and compression of adjacent spinal cord accompanied by tuberculosis of the adjacent appendix.

**Figure 5:**
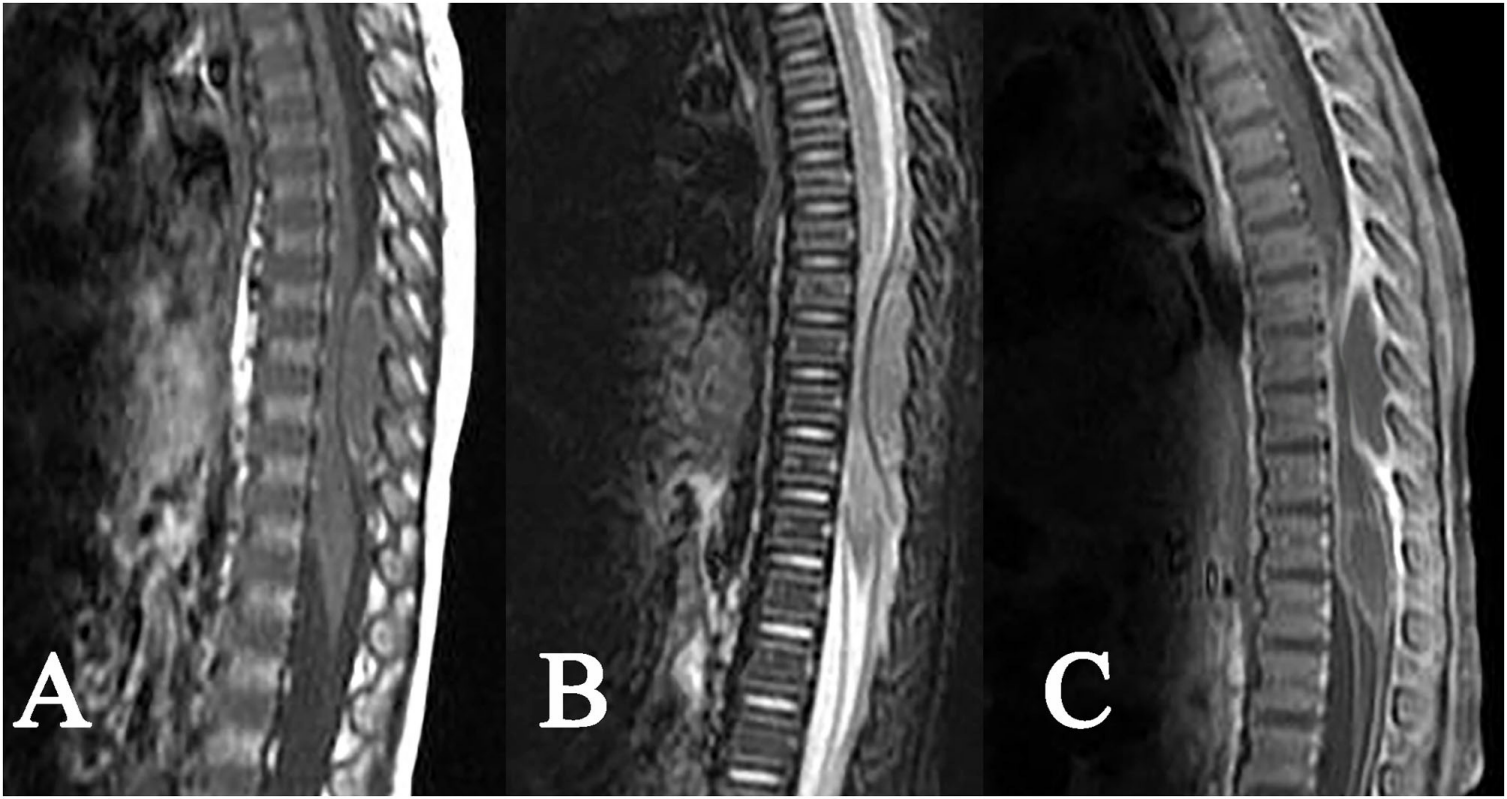
Epidural tuberculoma of the thoracic spinal cord. A fusiform lesion: **(A)** slight hypointense on T1WI, **(B)** isointense on T2WI, **(C)** rim enhancement after contrast enhancement, showing remarkable compression of the adjacent spinal cord and dural sac.

The epidural tuberculoma was located in the cervical spinal cord in 3 cases, in the thoracic spinal cord in 2 cases, in the lumbar spinal cord in 1 case, in the cervicothoracic junction in 1 case, and in the thoracolumbar junction in 3 cases. The lesions with long-fusiform or en plaque shapes showed a longitudinal course along the posterolateral or posterior side of the spinal cord. The longitudinal length of the lesions covered 2–7 vertebrae. The dural sac and spinal cord at corresponding segments were subjected to different degrees of compression. The MRI signals were characterized by slight hypointensity or isointensity on T1WI (Figures 4A, 5A), slight hypointensity, isointensity, or slight hyperintensity on T2WI (Figures 4B, 5B) and marked homogeneous (Figure 4C) or rim enhancement (Figure 5C), as shown in Table 1.

### The Association of Intraspinal Tuberculomas With Tuberculosis at Other Sites or an Antituberculosis History

Five cases of intramedullary tuberculoma were accompanied by multifocal tuberculosis, indicating the probability of hematogenous dissemination. One case of intradural extramedullary tuberculoma was complicated with tuberculous meningitis and diffused the enhancement signs of the spinal meninges, which were considered to be caused by cerebrospinal fluid dissemination. Additionally, 8 cases of epidural tuberculoma were accompanied by tuberculosis of adjacent vertebra or the appendix, suggesting that the lesion was induced by invasion of adjacent active tuberculosis. Furthermore, 10 cases of tuberculoma (such as 1 case of intramedullary tuberculoma, 7 cases of intradural extramedullary tuberculoma, and 2 cases of epidural tuberculoma) occurred during standard treatment for pulmonary tuberculosis or tuberculous meningitis with antituberculosis drugs (Table 2), indicating that the occurrence of these intraspinal tuberculomas might be associated with the “paradoxical response” mechanism during antituberculous treatment.

**Table 2:** The incidence of intraspinal tuberculoma during antituberculous therapy among the three groups.

	**Group 1 [*n* (%)]**	**Group 2 [*n* (%)]**	**Group 3 [*n* (%)]**	**Total [*n* (%)]**
**Occurrence during antituberculous therapy**				
Yes	1 (16.7%)	7 (87.5%)	2 (20%)	10 (41.7%)
No	5 (83.3%)	1 (12.5%)	8 (80%)	14 (58.3%)
*p* ^a^	1-2-3	1-2	1-3	2-3
	**0.007**	**0.026**	1.000	**0.015**

*Group 1: Intramedullary tuberculoma.*

*Group 2: Intradural extramedullary tuberculoma.*

*Group 3: Epidural tuberculoma.*

a *Fisher's exact test.*

*Statistically significant values are shown in bold*.

### Surgical Pathology

A total of 18 patients underwent surgical treatment. The lengths and specific locations of the lesions during surgery were basically identical to those determined by MRI. There were 12 cases of obvious adhesion of the lesion with the spinal dura mater and/or arachnoid, including 6 cases of nerve root adhesion. To avoid further injury, only the major portion of the lesion in patients with obvious adhesion was resected during operation, while the adhered portion was not removed. According to the pathology report, the lesion tissues appeared grayish-white, loose, and soft. Under a microscope, the intramedullary tuberculoma mainly showed caseous necrosis surrounded by granulation tissue in which epithelioid cells accumulated. Most of the extramedullary tuberculomas presented as inflammatory granuloma containing lots of epithelial cells and a small number of lymphocytes and fibroblasts. Caseous necrosis to various degrees was also observed in the lesion center in 7 cases of extramedullary tuberculoma.

## Discussion

As an infectious granulomatosis due to the invasion of MTB into the spinal cord and/or spinal meninges, intraspinal tuberculoma has a complicated pathogenesis. Pathogenic bacteria are generally from one of the following sources: (1) primary lesions disseminating *via* the blood, (2) lesions in the intracranial cavity or the spinal cord spreading through cerebrospinal fluid, or (3) tuberculosis infection directly invading adjacent sites. In the present study, 5 cases of intramedullary tuberculoma were complicated with multiple tuberculosis infections, indicating an infection route of hematogenous dissemination. In 1 case of intradural extramedullary tuberculoma, tuberculous meningitis, and diffuse enhancement of the spinal meninges were found, suggesting cerebrospinal fluid dissemination. In addition, 8 cases of epidural tuberculoma were accompanied by tuberculosis of adjacent vertebra or the appendix, which may have been caused by the invasion of adjacent active tuberculosis. In the other 10 cases, the infection route was unclear, but notably, these cases all occurred during antituberculous therapy, most of which were intradural extramedullary tuberculoma. This finding is in line with the findings reported in the literature that some patients with intradural extramedullary tuberculoma have a history of antituberculosis treatment. It is inferred that this finding may be associated with the so-called “paradoxical response” during treatment with antituberculosis drugs (22–26). The “paradoxical response” is a phenomenon of lesion exacerbation in some patients during antituberculous therapy. In terms of its specific mechanism, delayed-type hypersensitivity is mostly considered. In the case of active tubercle bacillus in the body, immune suppression occurs, which is relieved after antituberculous therapy. On the other hand, MTB killed by chemotherapy drugs is dissolved and releases proteins, with the latter serving as antigens to trigger cellular immune responses in the body, resulting in inflammatory responses dominated by lymphopoiesis. As a result, original occult lesions colonized on the spinal cord or meninges become larger to eventually form obvious tuberculomas (25). In this study, the incidence of intradural extramedullary tuberculoma occurring during antituberculous therapy was significantly higher than that of the other 2 types of intraspinal tuberculoma, possibly because the subdural structures have a rich blood supply and direct contact with cerebrospinal fluid, facilitating MTB colonization, and subsequent formation a latent lesion through hematogenous or cerebrospinal fluid-borne routes during the initial tuberculosis infection. Generally, intraspinal tuberculoma has an occult onset and a relatively slow progression, so lesions often have become very conspicuous when first detected. Lesions that are discovered late are more prone to adhering to the spinal meninges or nerve roots, increasing the difficulty of surgical resection and often leading to a poor prognosis. For this reason, once symptoms of spinal cord compression are observed after or during antituberculous therapy, high vigilance should be put on intraspinal tuberculoma, and MRI examination should be conducted in a timely manner. If available, patients can be recommended for regular MRI examination during the antituberculous therapy to discover lesions early even if they do not have corresponding clinical manifestations.

The MRI characterized by high soft tissue resolution and multidirectional and multiparameter imaging is particularly well-suited for displaying the location, shape, and signal features of intraspinal lesions, which is of great significance for guiding surgery (27–29). According to related literature (17, 19, 21) and cases in this study, intraspinal tuberculomas have the following MRI features: (1) Morphologically, intramedullary tuberculomas are mostly round or round-like, whereas extramedullary tuberculomas have largely long-fusiform or plaque-like shapes and show a longitudinal course along the spinal cord. Such morphological characteristics of intradural extramedullary and epidural tuberculomas may have correlations with the narrow extramedullary space in the spinal canal, the orientation of the meninges, and the soft texture of the lesions. (2) Intraspinal tuberculomas display diverse MRI signals, and both intramedullary and extramedullary lesions are mainly isointense on T1WI and hypointense or isointense on T2WI. MRI signal features reflect the tissue components of tuberculomas in different stages. A newly formed tuberculoma mainly consists of new tuberculous granulation tissues, with uniform isointensity on T2WI in most cases. As the lesion develops, caseous necrosis may appear in the tuberculoma, with hypointense (hyperintense or mixed signal if accompanied by liquefaction necrosis) on T2WI. In our series, caseous necrosis was less common in extramedullary tuberculomas than in intramedullary tuberculomas, possibly because extramedullary tuberculomas were more likely to cause symptoms of nerve root compression and thus more likely to be detected early. (3) Diverse enhancements were noted on contrast-enhanced scans, with rim enhancement in intramedullary lesions and obvious homogeneous enhancement in extramedullary lesions in most cases. Tuberculous granulomas typically have a rich blood supply and often exhibit marked enhancement. When necrosis occurs in lesions, rim enhancement is frequently observed on contrast-enhanced MRI images. In contrast with plain scans, contrast-enhanced scans can display the scope and shape of lesions as well as their associations with surrounding structures more clearly. Hence, contrast-enhanced scans are necessary in MRI examinations of the spinal canal.

Although intraspinal tuberculomas have certain MRI features, they should also be distinguished from other intraspinal space-occupying lesions: intraspinal tumors, bacterial granulomas or abscesses, and paragonimus granulomas. Regarding intraspinal tumors, astrocytomas and ependymomas are two main intramedullary tumors in children. These tumors do not have the typical characteristics of tuberculomas, i.e., hypointensity on T2WI. In addition, these tumors mostly show heterogeneous enhancement, and the rim wall is often irregular even if rim enhancement is observed (30, 31). Epidural neurogenic tumors are common among extramedullary tumors and are characterized by “dumbbell-shaped” changes across the intervertebral foramina. The MRI signals and enhancement patterns of these tumors are also different from those of tuberculomas (32). Lymphomas can also infiltrate into the spinal canal, with homogeneous signals on plain and contrast-enhanced scans. However, most lymphomas display mild-moderate enhancement, and the enhancement degree is inferior to that of tuberculomas. Moreover, intraspinal lymphomas are often accompanied by lymphoma manifestations in other parts (33). Bacterial granulomas or abscesses can also occur in the intraspinal canal. Their morphology and enhancement pattern are similar to those of tuberculomas, but the signal is usually hypointense on T1WI and hyperintense on T2WI unlike that of tuberculomas (34). Paragonimus granulomas are mostly found outside the dura mater and in the thoracic segment of the spine. These lesions are characterized by a tendency to bleed, which is easily detected on MRI. Another imaging feature of paragonimus granuloma is the attachment of epidural lesions to pleural or intrapulmonary lesions through the intervertebral foramina (35).

There are some limitations in this study. First, due to the rarity of intraspinal tuberculoma, the sample size in this study was relatively small. In particular, only 5 cases of intramedullary tuberculoma were enrolled in this study, the MRI signal patterns of which were relatively simple and may only reflect the histological characteristic of tuberculoma in a certain course and cannot represent the whole development and progression of tuberculoma. Second, in this retrospective study, some patients did not have follow-up MRI scans after lesions were found. The dynamic changes in MRI signals as the disease progresses are not known. In addition, MRI was conducted using only conventional sequences, and special sequences, such as diffusion-weighted imaging, were not performed. The application of these special sequences not only is conducive to increasing MRI spectra of intraspinal tuberculoma but can also help improve the accuracy of MRI in the diagnosis of the disease.

## Conclusions

In summation, MRI scans, especially contrast-enhanced MRIs, are capable of clearly displaying the scope, shape, and signal characteristics of lesions and are of measurable value for the diagnosis of intraspinal tuberculoma. Intraspinal tuberculoma shows diversiform MRI signals, with isointense on T1WI and isointense or hypointense on T2WI in most cases. In contrast-enhanced scans, intramedullary tuberculoma mostly displays rim enhancement, while extramedullary tuberculoma largely shows homogeneous enhancement. These MRI features will be helpful in the differentiation of intraspinal tuberculoma from other intraspinal lesions. In addition, some intraspinal tuberculomas, especially intradural extramedullary tuberculomas, occur during antituberculous therapy, which might be associated with the paradoxical response of the body to antituberculous drug treatment. Hence, once the symptoms of the spinal cord or spinal nerve root compression are encountered during antituberculous therapy, intraspinal tuberculoma should be highly suspected, and MR examination should be conducted in time. If available, MRI examination can be performed regularly to monitor the patients receiving antituberculous therapy and detect intraspinal lesions early.

## Data Availability Statement

The raw data supporting the conclusions of this article will be made available by the authors, without undue reservation.

## Ethics Statement

The studies involving human participants were reviewed and approved by Institutional Ethics Committee of Children's Hospital Affiliated to Chongqing Medical University. Written informed consent from the participants' legal guardian/next of kin was not required to participate in this study in accordance with the national legislation and the institutional requirements.

## Author Contributions

HZ, YQ, and JC designed the study. HZ and YQ collected the materials, analyzed the data, and wrote the manuscript. TM and HZ analyzed the data. JC revised the manuscript. All authors contributed to the article and approved the submitted version.

## Funding

This study was partly supported by a Grant from the National Natural Science Foundation of China (81171387).

## Conflict of Interest

The authors declare that the research was conducted in the absence of any commercial or financial relationships that could be construed as a potential conflict of interest.

## Publisher's Note

All claims expressed in this article are solely those of the authors and do not necessarily represent those of their affiliated organizations, or those of the publisher, the editors and the reviewers. Any product that may be evaluated in this article, or claim that may be made by its manufacturer, is not guaranteed or endorsed by the publisher.

## Abbreviations

MTB: mycobacterium tuberculosis
CNS: central nervous system
MRI: magnetic resonance imaging
T1WI: T1-weighted imaging
T2WI: T2-weighted imaging
TR: repetition time
TE: time of echo.
